# Value co-creation in business-to-business context: A bibliometric analysis using HistCite and VOS viewer

**DOI:** 10.3389/fpsyg.2022.1027775

**Published:** 2023-01-11

**Authors:** Fawad Ullah, Lei Shen, Syed Hamad Hassan Shah

**Affiliations:** ^1^Glorious Sun School of Business and Management, Donghua University, Shanghai, China; ^2^School of Business, Nanjing University, Nanjing, China

**Keywords:** Web of Science, VOS viewer, bibliometric review, HistCite, value co-creation, B2B

## Abstract

**Abstract purpose:**

Value co-creation (VCC) recently displayed a significant increase in the frequency of publications in business studies and social sciences. Our study objects to explore the current state of VCC research in the business-to-business (B2B) context, principally in the marketing field.

**Research design, approach, and methodology:**

This research article extracted research papers on VCC in the B2B context published in the last two decades through the Web of Science (WoS). Initially, we applied HistCite to determine the research dynamics of VCC articles and then VOS viewer to conduct bibliographic coupling and cartographic analysis. Furthermore, we found the most co-occurred keywords in the abstracts, titles, and keywords.

**Findings:**

Our research explored that the United Kingdom was the most important country with 27 publications and 594 citations. Aarikka-Stenroos L was the most influential author, among his research is a systematic review which revealed that scholars of B2B journals adopted the term business “ecosystem” and studied the implications of ecosystem perspective in business and innovation networks and received the most citations. Industrial Marketing Management (IMM) was the most influential journal because it published 8 of the 10 most cited articles. One hundred and six out of 121 publications were in Business research and seventy-six were in management area, which made it the most hot and critical research area. Lappeenranta University was the most essential organization in VCC research based on the most records published and second-highest citations.

**Research limitations/implications and future research:**

Four research streams have emerged which indicate the prominent role of VCC in the B2B context (1) VCC and relationships, (2) VCC and organizational capabilities, (3) VCC and actors’ engagement at various platforms, and (4) VCC and processes. Our research paper provided a base for conceptualizing publications related to business, management, operations research management science, and social sciences interdisciplinary on VCC in the B2B context. Content analysis has revealed that research work on VCC in the B2B context is at an early stage in the marketing arena. Along with bringing some sort of consensus regarding researchers’ opinion toward the nature and modality of VCC literature and process in the B2B context, we urge future research to focus on how relationships and their precursors can be efficiently utilized to co-create and enhance value within B2B interactions. We also request future research to focus on making the VCC process sustainable and viable both on a time and economical basis.

**Practical implications:**

Organizations can involve customers and producers to work jointly to co-create value for their goods and services with negligible cost to achieve higher market shares and a competitive edge over rivals.

**Originality/value:**

This might be the first bibliometric study conducted on VCC in the B2B context (there are some Bibliometric VCC publications, but they are not B2B-specific, our research is the first Bibliometric study conducted on VCC in the B2B context) in the marketing field and can expose novel avenues for future research.

## Introduction

Value is probably an elusive and the most ambiguous concept in services marketing and management (Carù and Cova, 2003; Woodall, 2003). Yet, various efforts were made to establish holistic conceptualizations of value (Khalifa, 2004), mainly on the individual level (Holbrook, 1999), ignoring the multiple stakeholder interactions. While investigating VCC, especially co-production as a construct that existed before 2000 in disciplines outside marketing Ranjan and Read (2016), co-creation research emerged through the studies of Prahalad and Ramaswamy (2004a) service-dominant logic that evolved in the new millennium post-2000. Apriliyanti and Alon (2017) have argued that it is imperative and necessary to conduct a bibliometric analysis every 5 years or at least once in 10 years so that emerging constructs and research streams are highlighted. Current studies consider and align CB (Customer Based) VCC processes with several stakeholders, mainly focusing on CB relationships (Cova et al., 2011; Merz et al., 2018). Very rarely have studies tried to investigate how CB value is co-created in an industrial environment (i.e., B2B, Business-to-Business) (Gupta et al., 2017; Törmälä and Saraniemi, 2018; Iglesias et al., 2020). For this purpose, we conducted this study and used co-citation analysis via HistCite to achieve a thorough understanding of VCC research, especially in marketing; with cartography analysis through VOS viewer, we exposed novel research streams to achieve a better understanding of current research.

According to Sarkodie and Strezov (2019), researchers mainly used HistCite and VOS Viewer in their bibliometric studies. Co-citation analysis is used as a meta-analytical tool in bibliometric studies to explore and demonstrate the interconnections among topics and research articles (Kim and McMillan, 2008) by examining how frequently articles cite a research article. HistCite also shows novel and vital research dynamics (most influential journals, authors, institutions, countries, and cited references) of a topic under consideration (Luukkonen, 1997; Nederhof and Satta, 2006). A research field’s genealogical antecedent could be explored swiftly through citation behavior because co-citation analysis highlights the most commonly cited articles (Fetscherin, 2010). We conducted co-citation analysis through HistCite, bibliographic coupling, and cartographic analysis through VOS Viewer to discover essential keywords in the VCC research stream.

At last, this research has attempted to answer the following research questions through co-citation, cartography, bibliographic coupling, and content analysis. The principal research questions lectured in the study are as follows:

RQ1. Which are the most critical and influential channels (institutions, countries, authors, journals, and articles) in VCC in the B2B context literature?

RQ2. How are VCC in B2B publications clustered. Which are the most emerging research streams in marketing studies?

RQ3. Which research streams in VCC received the utmost attention?

RQ4. Which guidelines will expose new avenues for future research in VCC?

## Theoretical background

### Emergence of value co-creation in business-to-business

According to Ranjan and Read (2016), VCC investigations have revealed that co-production as a construct existed before 2000, while co-creation construct emerged through service-dominant logic (Prahalad and Ramaswamy, 2004a). McColl-Kennedy et al. (2012) have revealed that 22 out of 27 definitions of co-creation happened after 2000, while the remaining were specific to co-production. Value is perhaps the most ambiguous concept (Carù and Cova, 2003; Woodall, 2003). Yet, various efforts were made to establish holistic conceptualization of value (Khalifa, 2004). Co-creation happens when consumers collaborate with product/service providers to create value (Prahalad and Ramaswamy, 2004b). According to Grönroos et al. (2000), Vargo and Lusch (2004), the value gets more embedded in co-creation when customers transform from passive to more active audiences. Ramaswamy (2009) pointed out that organizations should involve employees in the co-creation of customer value; it must ensure employees’ interaction with customers. In the light of above-mentioned definitions, our study has defined VCC as a two-way process in which manufacturers and customers work together to co create and enhance the value of their products and services.

Prahalad and Ramaswamy (2004a) challenged Porter Michael (1985) traditional and conventional value chain concept; they argued that it did not consider the customer’s role in the process of VCC. Prahalad and Ramaswamy (2004b) proposed the DART Model, which provides the basic foundation by suggesting four necessary conditions for VCC: dialog, access, risk reduction, and transparency. Smith and Colgate (2007) proposed a value-creation strategies framework that classified value into four categories in literature: cost/sacrifice, experiential/hedonic, functional/instrumental, and symbolic/expressive. Payne et al. (2008) proposed a process-based framework that identified three significant processes (steps) in the VCC, first customer value-creation, second, supplier value-creation, and third the encounter process.

Web 2.0 allowed interactions of firms with customers, which reinforced VCC at the customer’s end (Bell and Loane, 2010). Novel technology’s infusion in customer–firm interactions redefined the customer’s role in innovation and value creation (Bitner et al., 2000; Dahan et al., 2001; Thomke and Von Hippel, 2002). In general, current studies have considered and aligned customer-based (CB) VCC processes with several stakeholders, mainly focusing on CB relationships (Cova et al., 2011; Merz et al., 2018). Rare studies have investigated how CB value is co-created in the industrial environment (i.e., B2B, Business-to-Business) (Gupta et al., 2017; Törmälä and Saraniemi, 2018; Iglesias et al., 2020). Yet, B2B and B2C do not represent all existing markets or business places; they can also function in another environment B2B2C (Business-to-Business-to-Consumer), which synchronously consider both consumers and industrial organizations as customers (Iankova et al., 2019). With the increasing attention of practitioners’ Accenture (2014, 2016), and research scholars of marketing Brotspies and Weinstein (2019), Iankova et al. (2019), we don’t know how value is co-created in CB multiple stakeholders (B2B2C) markets. Furthermore, Iankova et al. (2019) believe “a treasure of research opportunity exists in the business models of B2B2C, and we know very little regarding the complementary of working in the traditional B2C and B2B sectors, which may yield novel insights.”

### The emergence of HistCite and VOS viewer

According to Zupic and Čater (2015), HistCite use a robust quantitative technique to analyze literature reviews systematically. Pan et al. (2018) investigated 481 research articles through VOS Viewer and HistCite for bibliometric mapping. According to Apriliyanti and Alon (2017), 336 articles followed HistCite for co-citation purposes, and 2088 articles used VOS Viewer for cartographic analysis to segregate literature for absorptive capacity into five research streams.

By following bibliometric analysis, researchers can swiftly identify a specific concept’s current state, significance and origin (Fellnhofer, 2019). Literature intensive examination has revealed that VOS viewer and HistCite are extensively used; hence, we followed both. HistCite is primarily used for bibliometric analysis to generate chronological tables which provide information about essential authors, institutions, countries, journals, and cited references (Levitt and Thelwall, 2008). Meanwhile, VOS Viewer is frequently used in bibliometric studies, mostly in thematic, cartographic, and cluster analysis (Llanos-Herrera and Merigo, 2019; Noor et al., 2020c). While using VOS viewer, researchers can promptly investigate and analyze bibliometric networks such as authors, publications, countries, organizations, and journals (van Eck and Waltman, 2017).

Bibliometric analysis has the following five ways, i.e., bibliographic coupling, cartographic analysis, co-occurrence of keywords, co-citation, and co-author. Olczyk (2016) used VOS viewer and HistCite to study and identify growth patterns in international competitiveness literature through bibliometric citation data (1945−2015) of the Web of Science (WoS). VOS viewer uses the text-mining method for keyword analysis to extract keywords content, abstracts, and titles to assist us in finding clusters of closely associated items. The significance of the items demonstrated depends upon the item’s size; a larger item size means higher significance (Perianes-Rodriguez et al., 2016).

## Methods

Our research is stirred from the research work of Vargo (2011), O’Cass and Viet (2012), Shah et al. (2019), González-Torres et al. (2020). This study also follows the methodology used by Shah et al. (2021a). The methodology was used in studies to conduct bibliometric analyses for specific research areas such as brand personality (Llanos-Herrera and Merigo, 2019), Twitter (Noor et al., 2020b,d), music therapy (Li et al., 2021), social media role in knowledge management (Noor et al., 2020a), maping trends in Moyamoya Angiopathy research (Chen et al., 2021), autism research (Whyatt and Torres, 2018), bibliometric analysis of scientific publications (Chriki et al., 2020), trends of postpartum depression (Bai et al., 2021), and podocyte injury research (Liu et al., 2021). It was also used in the marketing field to study its different aspects like global branding (Chabowski et al., 2013), global value chain (González-Torres et al., 2020), Prosumption (Shah et al., 2020), business-to-business branding (Seyedghorban et al., 2016), and VCC (Alves et al., 2016; Shah et al., 2021a).

**FIGURE 1:**
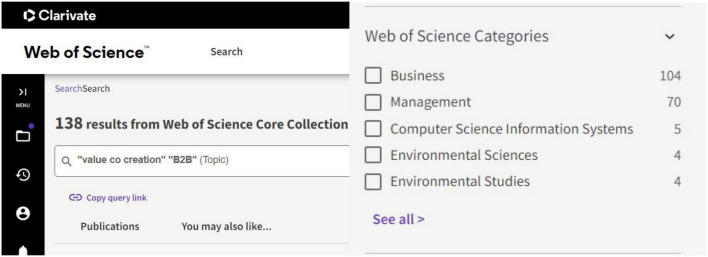
Total publications. Reproduced with permission of Clarivate.

This article searched “value co-creation” and “B2B” as keywords in the WoS, a top-quality database, and found 138 results, as shown in Figure 1 (September 2022). We selected 121 publications from business, management, operations research management science, and social sciences interdisciplinary. WoS is used for searching and tracking top journals of social science, arts, humanities, and basic science (Fetscherin and Heinrich, 2015). It has access to more than 50 million research publications in 70 languages, 151 categories, and 22,000 journals (Merigó et al., 2015). We preferred WoS over SciELO, Google Scholar, SCOPUS, and other search engines based on its competitiveness and quality.

About 4,409 studies used PLS-SEM (partial least square, structural equation modeling) in WoS core collection (October 2022), the most prominent studies were conducted by Henseler et al. (2015) who assessed the discriminant validity in variance-based structural equation modeling and Hair et al. (2011) who studied the use of PLS-SEM in the field of marketing. SEM is a technique used to study, measure, and analyze the relationship between or among observant and latent variables. Another important study was by Hair et al. (2019), they discussed when and how to use and report and interpret the results of PLS-SEM. About 115 studies on VCC within WoS core collection used SEM, among which 98 (85%) were published post-2017. While among the studied conducted in VCC in the B2B context, only seven research papers have used SEM.

Among the 121 VCC-B2B publications, 100 were articles, 10 were literature reviews, 6 were early access articles, 3 were editorial material and 1 were proceeding papers and corrections each.

**FIGURE 2:**
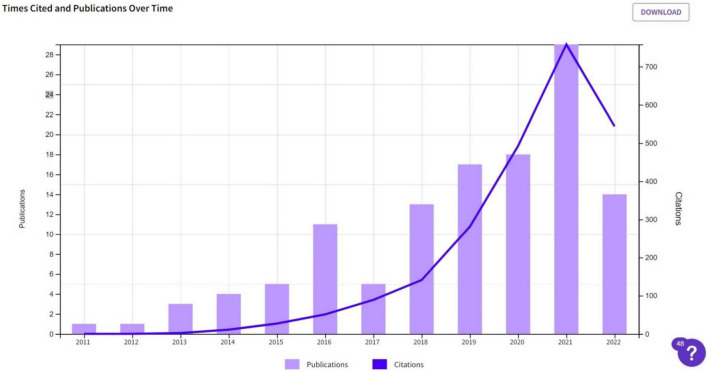
Citations and publications per year. Reproduced with permission of Clarivate.

The 121 results showed 1880 total citations at an average of 19.81 per year, as shown in Figure 2. The graph has shown an increasing trend in annual citations specifying the importance and emergence of research work in VCC. During cartographic analysis, we chose co-occurrence for the type of analysis and all keywords for the unit of analysis.

### Methods and analytical tools

**FIGURE 3:**
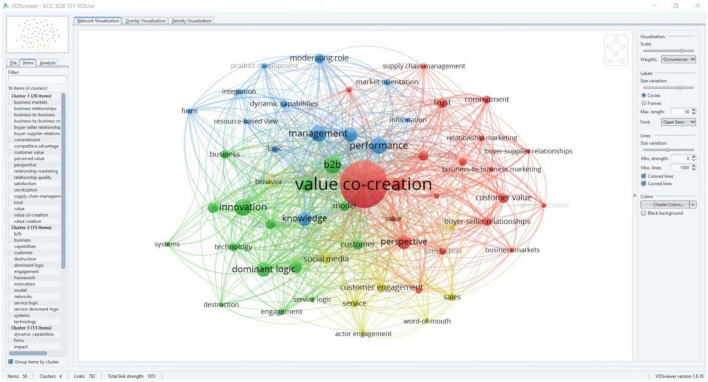
Cartography analysis using VOS viewer.

We searched “HistCite” and “VOS Viewer” in the core collection of WoS (September 2022). One hundred forty-six bibliometric studies used HistCite; 109 (74.65%) were from the last 5 years. Two hundred thirty-four studies used VOS Viewer, of which 221 (94.44%) were from the last 5 years, which illustrates its emergence and significance in contemporary bibliometric studies. In the bibliographic coupling analysis, the level and degree of authors citing the same research papers show the connection between their research. The nature of relatedness between unrelated authors depends on the number of times they cite a specific article; the higher the citation means higher the relatedness (Waltman et al., 2010). Unlike HistCite numerical representation in its citation graph, VOS Viewer can highlight author names and publication years. We can also use it to cluster publications based on a similar theme, topic, or field (Ding et al., 2001). Hence, with cartographic analysis, research streams of VCC were confirmed, as shown in Figure 3.

### The procedure of analysis of data

We extracted 121 papers from WoS published on VCC in the B2B context and saved them in a text document correctly to be used by HistCite later (September 2022). The document possessed the article’s titles, document’s type, abstract, authors and journals names, and language used. Then, we exported that file to HistCite and processed it to extract the most influential authors and journals names, articles, language, document types, countries, and institutes names. Furthermore, we performed a VCC citation mapping analysis to obtain a tabular form output (Garfield et al., 2003). Afterward, we took the services of VOS Viewer to conduct bibliographic coupling and cartographic analysis. The tabular and visual data were beneficial for assisting us in sighting and discovering the future research streams of VCC in the B2B context.

## Discussion and findings

### Value co-creation articles analysis through HistCite

#### Influential authors, articles, and journals

This study discovered that 339 researchers authored 121 articles. TGCS was the formula for sorting out the most influential and renowned authors. TGCS demonstrates the number of times articles reference a specific article globally. The total local citations score (TLCS) shows the number of times an article is referenced within a retrieved set of articles. The most prominent author was Aarikka-Stenroos L, with 193 TGCS, 6 TLCS score, and three records (Table 1). Second on the list was Ritala P, with 150 TGCS. Zolkiewski J with 138 was spotted third, Breidbach CF and Maglio PP (Breidbach and Maglio, 2016) with 127 TGCS were listed fourth and fifth, respectively. Baumann J and Keranen J were listed sixth and seventh. Chowdhury IN and Gruber T with the same TGCS and TLCS were placed eighth and ninth. Despite being listed at tenth Rajala R is still an essential author for showing the highest TLCS among the top 10 authors as shown in Table 1.

**TABLE 1:** Most influential authors.

#	Author	Recs	TLCS	TGCS
1	Aarikka-Stenroos L	3	6	193
2	Ritala P	1	0	150
3	Zolkiewski J	2	13	138
4	Breidbach CF	1	14	127
5	Maglio PP	1	14	127
6	Baumann J	2	4	115
7	Keranen J	4	5	109
8	Chowdhury IN	1	10	102
9	Gruber T	1	10	102
10	Rajala R	2	26	101

The article published by Aarikka-Stenroos L and Ritala P was on top, with a TGCS score of 150. The runner-up study was Breidbach and Maglio (2016) with 127 TGCS. We ranked Chowdhury et al. (2016) third with a 102 TGCS score. The study conducted by Marcos-Cuevas et al. (2016) was placed fourth with a TGCS score of 94 and Kohtamäki and Rajala (2016) was placed fifth. Articles of Bruhn et al. (2014) with 89 TGCS, Singaraju et al. (2016), Nguyen QA, Niininen O, and Sullivan-Mort G with 86 TGCS value and Hilton T, Hughes T, Little E, and Marandi E with 75 TGCS were ranked sixth, seventh, and eighth, respectively. The article of Hein A, Weking J, Schreieck M, Wiesche M, and Bohm M with 72 TGCS was listed ninth and Zhang et al. (2015) with 70 TGCS was tenth. Based on TLCS value Kohtamäki and Rajala (2016) showed the highest value of 25. From Table 2 our study also found that all the top ten research articles were published in one-decade post 2012, among which one was published in 2013, 2014, and 2015 each, five in 2016, one in 2017 and 2019 each. This shows the emergence and novelty of the topic.

**TABLE 2:** Most influential articles.

#	References	TLCS	TGCS	LCR	CR
1	Aarikka-Stenroos et al., 2017	0	150	2	141
2	Breidbach and Maglio, 2016	14	127	0	83
3	Chowdhury et al., 2016	10	102	0	102
4	Marcos-Cuevas et al., 2016	0	94	0	75
5	Kohtamäki and Rajala, 2016	25	92	2	76
6	Bruhn et al., 2014	1	89	0	116
7	Singaraju et al., 2016	6	86	0	92
8	Hilton et al., 2013	0	75	0	44
9	Hein et al., 2019	8	72	0	69
10	Zhang et al., 2015	8	70	0	133

We sorted the rankings of the journals based on the number of publications and the TGCS value. Our study found that the selected 121 research articles were published in 33 journals. As shown in Table 3, 41 out of 121 research articles were published in *IMM* having TGCS value of 1417, which shows the importance of *IMM* for its publications in the relevant research. The TGCS value of other journals was comparatively less because IMM published eight out of the top ten (most-cited) research articles. We also found that 31 research articles were published in the *Journal of Business and Industrial Marketing*, having a TGCS value of 278. Similarly, the *Journal of Business Research*, and *Journal of Marketing Management* showed four publications each, with 61 and 43 TGCS value were ranked third and fourth respectively. *European Journal of Marketing* and *Journal of Hospitality and Tourism Management* with the same publications were ranked fifth and sixth, respectively. Furthermore, all the remaining four journals showed two publications.

**TABLE 3:** Most influential journals.

#	Journal	Recs	TLCS	TGCS
1	Industrial Marketing Management	41	104	1417
2	Journal of Business and Industrial Marketing	31	28	278
3	Journal of Business Research	4	2	61
4	Journal of Marketing Management	4	8	43
5	European Journal of Marketing	3	5	43
6	Journal of Hospitality and Tourism Management	3	3	63
7	International Journal of Technology Management	2	3	8
8	Journal of Business-To-Business Marketing	2	3	19
9	Journal of Purchasing and Supply Management	2	1	18
10	Journal of Service Research	2	1	36

#### Most prominent institutions and countries

Our study found that 200 institutions participated in 121 publications. We sorted the top 10 most influential and prominent institutions in Table 4 based on publications and then TGCS value. We found the Lappeenranta University of Technology on top of the list with six publications and a TGCS value of 266. Aalto University showed five records and 165 TGCS. The University Valencia and Lebanese Amer University showed four publications each and were listed third and fourth, respectively. All the remaining six universities published three research papers each. Despite Aalto University being ranked second, it showed the highest TLCS value of 28.

**TABLE 4:** Most influential institutions.

#	Institution	Recs	TLCS	TGCS
1	Lappeenranta Univ. Technol.	6	6	266
2	Aalto Univ.	5	28	165
3	Univ. Valencia	4	0	21
4	Lebanese Amer Univ.	4	3	3
5	Georgia State Univ.	3	3	89
6	Free Univ. Berlin	3	4	35
7	Griffith Univ.	3	2	28
8	Aarhus Univ.	3	1	27
9	ESCP Business Sch.	3	0	24
10	Univ. Verona	3	1	9

A total of 37 countries were involved in publishing the 121 VCC in B2B research papers. We ranked the Countries based on the number of records published and then TGCS value; Table 5 shows the top ten countries publishing these 121 research papers. The United Kingdom was on top of the list with 27 publications and 594 TGCS values. The United States with 21 publications and a TGCS value of 385 was listed second. Finland with 18 publications and a TGCS value of 619 was ranked third. The people’s republic of China was fourth with 15 publications and a TGCS value of 217. Australia with 11 records was listed fifth. Sections “Influential authors, articles, and journals” and “Bibliographic coupling using VOS viewer” are an attempt to answer the research question (RQ1).

**TABLE 5:** Most influential countries.

#	Country	Recs	TLCS	TGCS
1	United Kingdom	27	31	594
2	United States	21	31	385
3	Finland	18	55	619
4	People’s Republic of China	15	14	217
5	Australia	11	26	309
6	Germany	9	22	214
7	France	8	9	139
8	Italy	8	7	98
9	Spain	7	0	60
10	India	7	8	50

### Research streams of 121 value co-creation research papers

#### Citation mapping analysis

**FIGURE 4:**
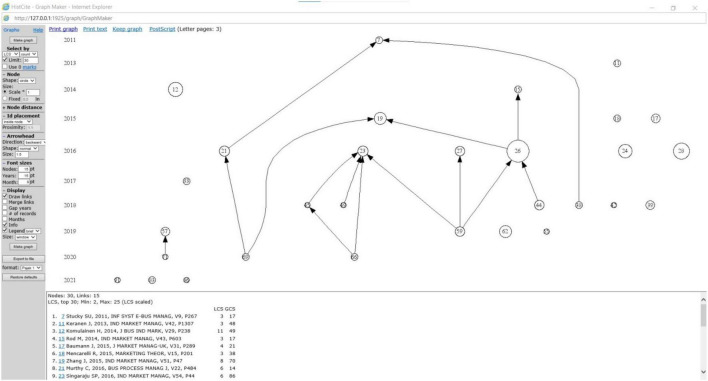
HistCite citation mapping analysis. Reproduced with permission of Clarivate.

**FIGURE 5:**
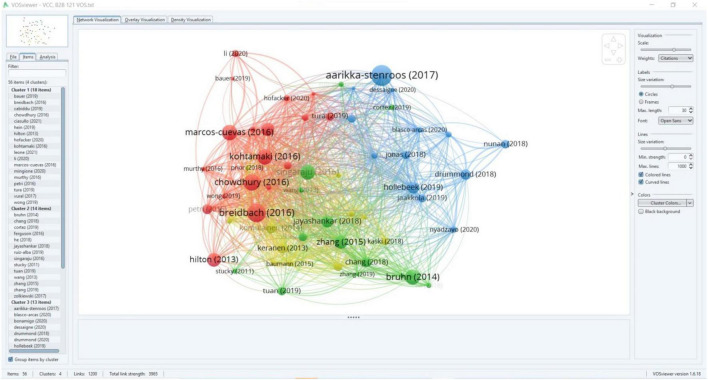
Bibliographic couplings using VOS viewer.

Figure 4 shows HistCite co-citation analysis. HistCite graph maker revealed 15 links (relationships among articles) from 30 articles (nodes) as the most cited. The graphs displayed the interconnection between these articles and how these articles have cited other articles. The citation mapping of HistCite also displayed the existing situation of all these 30 articles. It exposed two central and most crucial research articles, which were the epicenter of most of the connections among the top 30 articles. Kohtamäki and Rajala (2016) (number 26) on HistCite graph with 25 TLCS and 92 TGCS and Singaraju et al. (2016) (number 23) with six TLCS and 86 TGCS value as shown in Figure 5.

Based on TLCS value, the study by Kohtamäki and Rajala (2016) with 25 was ranked first, Breidbach and Maglio (2016) with 14 was spotted at second and Komulainen (2014) with 11 scores grabbed the third position. On the basis of TGCS value, the study by Breidbach and Maglio (2016) was first, Chowdhury et al. (2016) was second and Kohtamäki and Rajala (2016) was third with a score of 127, 102, and 92, respectively.

#### Bibliographic coupling using VOS viewer

In our study, bibliographic coupling was used for citation analysis. It offers a variety of analyses such as authors, journals, and publications (van Eck and Waltman, 2017). Circle size determines an article’s importance; a larger circle indicates more significance (van Eck and Waltman, 2010). During the analysis, for the type of analysis, we selected bibliographic coupling for the unit of analysis, we selected publications and full counting as the method in the VOS viewer. We selected a threshold value of 10 (citations) to bring rigor and precision to the analysis of VCC research streams. In total, we received 56 publications that emerged into four clusters, 1200 links, and a total link strength value of 3965. Represented by red color (having 18 articles) cluster 1 was the largest. The study by Breidbach and Maglio (2016) with 127 was the most cited article, while Chowdhury et al. (2016) with 102 the second, and Marcos-Cuevas et al. (2016) with 94 the third most cited article. Marcos-Cuevas et al. (2016) showed the highest links value of 52 while Chowdhury et al. (2016) showed the highest value of 288 for total links strength. Cluster 1 showed a total of 844 citations, 779 links, and total links strength value of 2657. Cluster 2 was represented by green color and possessed 14 articles and was the second largest. Bruhn et al. (2014) with 89 citations was the most cited article, Singaraju et al. (2016) with 86 was second and the Zhang et al. (2015) with 70 citations was the third most cited article in Cluster 2. Singaraju et al. (2016) showed the highest number of links, with 52, and Zhang et al. (2015) showed the highest value for total links strength. Cluster 2 showed 541 total citations and 1515 total links strength value.

Cluster 3 was represented by blue color containing 13 articles. Aarikka-Stenroos et al. (2017) showed 150 citations, Hollebeek (2019) with 62 was the second and Drummond et al. (2020) with 50 was the third most cited article in Cluster 3. Hollebeek (2019) displayed the highest value of links, with 52, and 230 total links strength. Cluster 3 showed 512 citations, 555 links and 1826 of total links strength value. Cluster four, represented by a yellow color, possessed 11 articles and was the smallest cluster. Komulainen (2014) showed the highest citation value of 49, Keränen and Jalkala (2013) with 48 was the second and Mencarelli and Rivière (2015) with 38 was the third most cited article in Cluster 4. La Rocca and Snehota (2014), Baumann and Le Meunier-FitzHugh (2015), Mencarelli and Rivière (2015), Park and Lee (2018) showed the highest links value of 50, while Baumann and Le Meunier-FitzHugh (2015) showed the total links strength value of 225. Cluster 4 showed 276 total citations, 513 links, and 1936 of total links strength value. Graphical representation and tabular explanation can be seen in Figure 5 and Table 6.

**TABLE 6:** Keywords co-occurrences in respective clusters.

Sr.no	Keywords	Co- occurrences	Links	Total links strength
**Cluster 1**				
1	Value co-creation	103	55	505
2	Buyer-supplier relations	27	17	27
3	Perspective	20	40	104
4	Business relationships	18	44	104
5	Customer value	12	30	66
6	Relationship quality	12	37	71
7	Trust	12	31	63
8	Buyer-seller relationship	10	28	55
9	Business-to-business	9	31	50
10	Commitment	9	23	45
11	Satisfaction	8	27	37
12	Value creation	7	24	37
13	Competitive advantage	6	24	34
14	Relationship marketing	6	21	35
15	Servitization	6	21	30
16	Supply chain management	6	21	30
17	Value	6	26	36
18	Business markets	5	18	28
19	Business-to-Business marketing	5	17	27
20	Perceived value	5	18	32
21	Total	270	553	1426
**Cluster 2**				
22	B2B	26	47	156
23	Innovation	26	41	155
24	Dominant logic	21	39	112
25	Service-dominant logic	20	41	110
26	Framework	16	35	93
27	Networks	13	30	68
28	Capabilities	12	34	65
29	Customer	12	30	72
30	Model	12	36	68
31	Business	10	23	45
32	Technology	10	32	68
33	Engagement	7	21	36
34	Destruction	5	15	73
35	Service logic	5	18	21
36	Systems	5	10	19
37	Total	200	452	1071
**Cluster 3**				
38	Performance	25	42	121
39	Management	24	47	139
40	Impact	21	45	113
41	Knowledge	20	40	109
42	Moderating role	12	28	59
43	Dynamic capabilities	8	21	39
44	Logic	7	25	44
45	Market orientation	7	24	35
46	Firms	6	21	35
47	Integration	6	22	33
48	Product development	6	21	29
49	Information	5	18	27
50	Resource-based view	5	18	25
51	Total	152	372	818
**Cluster 4**				
52	Customer engagement	11	34	68
53	Social media	11	31	61
54	Sales	9	31	56
55	Services	9	29	59
56	Actor engagement	5	22	33
57	Behavior	5	23	33
58	Loyalty	5	22	28
59	Word-of-mouth	5	15	22
60	Total	60	207	357

#### Cartography analysis through VOS viewer

By exploring the research streams of 121 VCC articles through bibliographic coupling analysis, we categorized research streams through the cartography analysis technique of VOS Viewer, as displayed in Figure 3. During the analysis process, a map was constructed for frequently occurring keywords in 121 VCC research papers. We selected co-occurrence for the type of analysis and all keywords for the unit of analysis. Furthermore, our study selected five as a minimum threshold for shared keywords to bring rigor and precision to the analysis. Consequently, we got 56 frequently occurring keywords in VCC in B2B research (Figure 3). VOS Viewer classified keywords into four main clusters and transferred them into visual form in the network visualization view. The size of the circle and label shows the significance of the keywords; a larger circle means more significance. Furthermore, keywords of the same color belong to the same cluster (van Eck and Waltman, 2010; Figure 3).

Among the total 56 keywords, Cluster 1 (marked in red) possessed 20 keywords. The keyword “value co-creation” had the highest co-occurrence value of 103, the highest links value of 55 and total links strength value of 505. Cluster 1 showed 270 total co-occurrences of keywords, 553 links, and 1426 total links strength value. Cluster 2, represented by green color was the second largest, and it possessed 15 keywords. “B2B” and “innovation” showed 26 co-occurrences each. Similarly, the keyword “B2B” showed the highest links value of 47 and total links strength value of 156. Cluster 2 displayed 200 co-occurrences of keywords, 452 links, and 1071 total links strength value. Cluster 3, shown in blue, possessed 13 keywords and was the third largest. Keyword “performance” showed the highest co-occurrences of 25, while the keyword “management” showed the highest links value of 47 and total links strength value of 139. Cluster 3 showed 152 total co-occurrences of keywords, 372 links, and 818 total links strength. Cluster four represented in yellow color was the smallest of all clusters and possessed 8 keywords. “Customer engagement” and “Social media” showed the highest co-occurrences value of 11 each. Keywords “Sales” and “Social media” showed 31 links while “customer engagement” showed 68 total links strength value. Keywords of cluster four exhibited 60 co-occurrences of keywords, 207 links, and 357 total links strength value. With the discussion in Sections “Citation mapping analysis,” “Bibliographic coupling using VOS viewer,” and “Cartography analysis through VOS viewer,” we have answered the research question (RQ2).

### Cluster analysis

We found 56 research articles that fulfilled the criteria of having at least 10 citations in 121 VCC in B2B publications. These were categorized into four clusters (Tables 7–10) having details about author names, titles, weighting (number of links and total link strength), and TGCS. The significance and importance of each research paper depend upon its weight in the particular cluster (Rodrigues et al., 2014; van Eck and Waltman, 2017). We followed two standard weighting features (total link strength and number of links), highlighting the article’s significance. In bibliographic coupling analysis, a link shows the connection or relation between two articles. Every link possesses a number value which explains its strength, which is higher when the link is stronger (van Eck and Waltman, 2017). VOS Viewer calculated links of specific articles and their strength values into one value of total link strength. We ranked the articles within the clusters based on the number of links and TGCS value. The higher the number of links and TGCS value, the higher the ranks. We ranked articles with similar links and TGCS values according to their respective total links strength value, the higher the total links strength value, the higher the ranking.

**TABLE 7:** Cluster 1.

Sr. No	Author	Title	Links	Total link strength	TGCS
1	Marcos-Cuevas et al., 2016	Value co-creation practices and capabilities: sustained purposeful engagement across B2B system.	52	192	94
2	Altuntas Vural, 2017	Service-dominant logic and supply chain management: a systematic literature review	52	209	120
3	Chowdhury et al., 2016	Every cloud has a silver lining − Exploring the dark side of value co-creation in B2B service networks	51	288	102
4	Breidbach and Maglio, 2016	Technology-enabled value co-creation: an empirical analysis of actors, resources, and practices	50	195	127
5	Kohtamäki and Rajala, 2016	Theory and practice of value co-creation in B2B system	50	216	93
6	Leone et al., 2021	How does artificial intelligence enable and enhance value co-creation in industrial markets? An exploratory case study in the healthcare ecosystem	50	123	26
7	Mingione and Leoni, 2020	Blurring B2C and B2B boundaries: corporate brand value co-creation in B2B2C markets	50	234	20
7	Cabbidu et al., 2019	Toxic Collaborations: Co-Destroying Value in the B2B Context	47	162	37
8	Petri and Jacob, 2016	Customers as enablers of Value Co-creation in solution business.	46	184	47
9	Wong and Lai, 2019	The effects of value co-creation activities on the perceived performance of exhibitions: A service science perspective	46	136	18
10	Ciasullo et al., 2021	A digital servitization framework for viable manufacturing companies	45	125	11
11	Hofacker et al., 2020	Digital marketing and business-to-business relationships: a close look at the interface and a roadmap for the future	44	104	21
13	Hein et al., 2019	Value co-creation practices in business-to-business platform ecosystems	42	105	72
14	Tura et al., 2019	The darker side of sustainability: Tensions from sustainable business practices in business networks	41	111	38
15	Murthy et al., 2016	An empirical investigation of the antecedents of value co-creation in B2B IT services outsourcing	40	112	14
16	Hilton et al., 2013	Adopting self-service technology to do more with less	39	84	75
17	Bauer and Borodako, 2019	Trade show innovations − Organizers implementation of the new service development process	17	20	11
18	Li et al., 2020	The state-of-the-art of the theory on Product-Service Systems	17	57	28

**TABLE 8:** Cluster 2.

Sr. No	Author	Title	Links	Total link strength	TGCS
1	Singaraju et al., 2016	Social media and value co-creation in multi stake holder system. A resource integration approach.	52	186	86
2	Chang et al., 2018	Enhancing firm’s performance: The role of brand orientation in Business-to-business marketing.	50	184	46
3	Jayashankar et al., 2018	Iot adoption in agriculture: the role of trust, perceived value, and risk.	50	150	62
4	Zhang et al., 2015	Building industrial brand equity by leveraging firm capabilities and co-creating value with customers.	49	215	70
5	Zhang and Zhu, 2019	When can B2B firms improve product innovation capability (PIC) through customer participation (CP)? The moderating role of inter-organizational relationships?	47	111	11
6	Zolkiewski et al., 2017	Strategic B2B customer experience management: the importance of outcomes-based measures	46	150	36
7	Ferguson et al., 2016	The social context for value co-creations in an entrepreneurial network Influence of interpersonal attraction, relational norms, and partner trustworthiness	41	84	46
8	Tuan et al., 2019	Customer value co-creation in the business-to-business tourism context: The roles of corporate social responsibility and customer empowering behaviors	39	70	38
9	Wang et al., 2013	Customer participation and project performance: the mediating role of knowledge sharing in the Chinese telecommunication service industry.	38	63	17
10	Bruhn et al., 2014	Antecedents and consequences of the quality in e-costumer-to- customer interactions in B2B brand communities.	35	92	89
11	Ruiz-Alba et al., 2019	Servitization strategies from customers’ perspective: the moderating role of co-creation	32	70	17
12	He et al., 2018	Influence of interfirm brand values congruence on relationship qualities in B2B contexts	29	67	17
13	Stucky et al., 2011	Dynamics of value creation in complex IT service engagements.	25	29	17
14	Cortez and Johnston, 2019	Marketing role in B2B settings: evidence from advanced, emerging and developing markets	20	42	20

**TABLE 9:** Cluster 3.

Sr. No	Author	Title	Links	Total link strength	TGCS
1	Hollebeek, 2019	Developing business customer engagement through social media engagement-platforms:	52	230	62
2	Jonas et al., 2018	Stakeholder engagement in intra- and inter-organizational innovation: Exploring antecedents of engagement in service ecosystems	48	161	35
3	Möller and Halinen, 2018	IMP thinking and IMM: Co-creating value for business marketing.	48	220	24
4	Blasco-Arcas et al., 2020	Organizing actor Engagement: A platform perspective	47	167	21
5	Laczko et al., 2019	The role of a central actor in increasing platform stickiness and stakeholder profitability: Bridging the gap between value creation and value capture in the sharing economy	47	108	34
6	Bonamigo et al., 2020	Facilitators and inhibitors of value co-creation in the industrial services environment	46	121	10
7	Jaakkola and Aarikka-Stenroos, 2019	Customer referencing as business actor engagement behavior − Creating value in and beyond triadic settings.	46	133	35
8	Drummond et al., 2018	The impact of social media on resource mobilization in entrepreneurial firms	43	128	50
9	Jaakkola and Aarikka-Stenroos, 2019	Customer referencing as business actor engagement behavior – Creating value in and beyond triadic settings	41	117	150
10	Drummond et al., 2020	Digital engagement strategies and tactics in social media marketing	41	138	21
11	Dessaigne and Pardo, 2020	The network orchestrator as steward: Strengthening norms as an orchestration practice	39	109	10
12	Nyadzayo et al., 2020	B2B purchase engagement: Examining the key drivers and outcomes in professional services	37	131	23
13	Nunan et al., 2018	Reflections on “social media: Influencing customer satisfaction in B2B sales” and a research agenda	20	63	37

**TABLE 10:** Cluster 4.

Sr. No	Author	Title	Links	Total link strength	TGCS
1	Baumann and Le Meunier-FitzHugh, 2015	Making value co-creation a reality, exploring the co-creative value process in customer-sales person interaction.	50	225	21
2	La Rocca and Snehota, 2014	Value creation and organizational practices at firm boundaries	50	177	19
3	Mencarelli and Rivière, 2015	Perceived value in B2B and B2C: a comparative approach and cross fertilization	50	219	38
4	Park and Lee, 2018	Early-stage value co-creation network − business relationships connecting high-tech B2B actors and resources: Taiwan semiconductor business network case	50	186	12
5	Pinnington et al., 2016	A grounded theory of value dissonance in strategic relationships	47	199	11
6	Komulainen, 2014	The role of learning in value co-creation in new technological B2B services	46	176	49
7	Keränen and Jalkala, 2013	Toward a framework of costumer value assessment in B2B markets; An Exploratory study.	46	215	48
8	Rod et al., 2014	Managerial perceptions of service infused IORs in China and India: A discursive view of value co-creation	44	180	17
9	Prior et al., 2018	Sense making, sense giving and absorptive capacity in complex procurements	44	130	14
10	Kaski et al., 2018	Rapport building in authentic B2B sales interaction	44	133	30
12	Taylor et al., 2020	value propositions in a digitally transformed world	42	117	17

#### Cluster 1 value co-creation and relationships

Cluster one possessed 18 articles as listed below, including the title, author’s name, links, total link strength, and TGCS score. Research articles authored by Marcos-Cuevas et al. (2016), Altuntas Vural (2017) showed 52 links each and were ranked first and second, respectively, while Chowdhury et al. (2016) with 51 links was listed 3rd. Breidbach and Maglio (2016), Kohtamäki and Rajala (2016), Mingione and Leoni (2020), Leone et al. (2021) displayed 50 links each and were ranked, respectively. Chowdhury et al. (2016) exhibited the highest value for total links strength 288 followed by Mingione and Leoni (2020) with 234 and Kohtamäki and Rajala (2016) with 216. Breidbach and Maglio (2016) showed the highest TGCS value of 127, Altuntas Vural (2017) showed 120 and Chowdhury et al. (2016) displayed 102, being the third. The ranking is given based on the links value and then in alphabetical order.

Altuntas Vural (2017) conducted a literature review of service-dominant logic in B2B marketing and supply chain management studies. This research identified five key research streams, (1) value-in-use and VCC, (2) integration and relationship management, (3) service supply chains, (4) resource sharing, and (5) Servitization. Chowdhury et al. (2016) focused on the dark side of VCC and studied how role conflict, role ambiguity, opportunism, and power impacted the VCC process negatively. Breidbach and Maglio’s (2016) empirical study focused on the information technology (IT) role in the co-creation of value in complex B2B service systems of consulting industry. Kohtamäki and Rajala (2016) reviewed the theories and methods used in VCC and co-production literature. They covered numerous viewpoints of economic and social exchange theory within multi-actor service ecosystems. Petri and Jacob (2016) termed customers as enablers. This cluster mostly focused on precursors (trust, commitment) and their outcomes (satisfaction, relationship quality and competitive advantage) in the relationships (buyer- seller, B2B, buyer- supplier relation, relationship marketing) and the resources needed for them in various industries (services and supply chain, etc.).

#### Cluster 2 value co-creation and organizational capabilities

Cluster 2 contains 14 articles, as shown in Table 8. Singaraju et al. (2016) showed 52 links and was first in the ranking. Chang et al. (2018), Jayashankar et al. (2018) showed 50 links each and were placed second and third. Zhang et al. (2015) displayed 49 links occupying the fourth place. Zhang and Zhu (2019) showed 47, Zolkiewski et al. (2017) showed 46, and Ferguson et al. (2016) showed 41 links and were ranked fifth, sixth, and seventh, respectively. Zhang et al. (2015) showed the highest links strength value of 215, followed by Singaraju et al. (2016), Chang et al. (2018) with 186 and 184, respectively. Bruhn et al. (2014) exhibited the highest TGCS value of 89, Singaraju et al. (2016) 86, and Jayashankar et al. (2018) 62.

Singaraju et al. (2016) reviewed the literature on the actor-to-actor (A2A) model and SD logic and studied the significance of social media platforms as system resource integrators between firms and customers interactions. Chang et al. (2018) revealed that marketing capability and entrepreneurial orientation are vital and positively affect a firm’s brand orientation, influencing brand performance by facilitating customer VCC activities. Jayashankar et al. (2018) studied the influence of trust in technology adoption by farmers in the agriculture sector. The study also revealed that perceived value is positively related to trust and negatively affiliated with perceived risk. Zhang et al. (2015) study unveiled that networking and marketing capability via customer value and VCC both, directly and indirectly, create brand equity, while innovation capability positively and indirectly impacts brand equity by enhancing and facilitating customer value and VCC.

This cluster mainly focused on how and which organizational capabilities can bring changes like innovation, the role of technology in increasing the value of service-dominant logic, and how to engage and utilize networks in today’s world of technology. It also discussed how all these factors influence value in B2B interaction in different industries.

#### Cluster 3 value co-creation in actor’s engagement at various platforms

Cluster 3 exhibited 13 articles with Hollebeek LD showing the highest links value of 52. Jonas et al. (2018), Möller and Halinen (2018) showed 48 links each and were placed second and third, respectively. Laczko et al. (2019), Blasco-Arcas et al. (2020) exhibited 47 links and were ranked fourth and fifth, respectively. Hollebeek (2019) showed the highest links strength value of 230, Möller and Halinen (2018) with 220 was second, and Blasco-Arcas et al. (2020) with 167 was third. Jaakkola and Aarikka-Stenroos (2019) showed the highest TGCS value of 150, followed by Hollebeek (2019) with 62, and Drummond et al. (2018) with 50.

Hollebeek (2019) focused on how important social media platforms and customer engagement are for businesses, and how can businesses engage their customers, while Jonas et al. (2018) studied the precursors of engagement and the role of stake holders’ engagement in intra- and interorganizational innovation within service ecosystems. This cluster focused on how to engage customers, Stakeholders, business actors, networks, purchasers, etc. It also studied how to enhance engagement at each level (social media and digital platforms, business platforms, and industrial environment). It also studied the role of management, knowledge, and information sharing in enhancing performance.

#### Cluster 4 value co-creation and processes

La Rocca and Snehota (2014), Baumann and Le Meunier-FitzHugh (2015), Mencarelli and Rivière (2015), Park and Lee (2018) all showcased 50 links and were ranked, respectively. Pinnington et al. (2016) showed 47 and was ranked fifth. Keränen and Jalkala (2013), Komulainen (2014) displayed 47 links each and were ranked sixth and seventh. Baumann and Le Meunier-FitzHugh (2015) showed the highest value for total links strength of 225 followed by Mencarelli and Rivière (2015) with 219 and Keränen and Jalkala (2013) with 215. Komulainen (2014) presented the highest TGCS value of 49, Keränen and Jalkala (2013) 48, and Mencarelli and Rivière (2015) 38.

Baumann and Le Meunier-FitzHugh (2015) explored the VCC process through in-depth interviews of salespersons and their customer interactions. During the co-creative interaction, both parties played different roles that were driven by characteristics, like sharing interests, establishing equitable dialog and commitment to common goal achievement. La Rocca and Snehota (2014) explored the organizational issues involved in implementing value programs in B2B firms and explored the required managerial actions. Keränen and Jalkala (2013) empirically studied the customer value assessment process from three solution suppliers’ perspectives. They proposed a five-step process framework (potential value identification, baseline assessment, performance evaluation, long-term value realization, and systematic data management). Mencarelli and Rivière (2015) compared the understanding of the perceived value in B2B and B2C spheres and adopted a micro-analytical (zoom-in) approach. Komulainen (2014) focused on customer sacrifices and motivation toward learning and involvement in VCC with the service provider. Section “Cluster analysis” provides an answer to the research question RQ3.

### Research guidelines for future studies

This research paper’s final objective was to propose guidelines for further studies, explore and advance VCC’s role in the marketing discipline. Our research paper followed Apriliyanti and Alon (2017), Shah et al. (2019) methodology using content analysis or the conventional literature review method. We proposed future research guidelines from research articles having comparatively high links and total links strength. We selected 17 research papers and their guidelines, eight from Cluster 1, four from Cluster 2, two from Cluster 3, and three from Cluster 4, as shown in Table 11.

**TABLE 11:** Future research guidelines.

Sr. No.	Future research guidelines	Authors
**Cluster 1**		
1	We urge future research to find out VCC precursors through empirical work to explore the conditions in which VCC can materialize.	Marcos-Cuevas et al., 2016
2	How to utilize data collected from other sectors to inspect if ambiguity and role conflicts, power plays, and opportunism are demonstrated as the same? Further longitudinal studies should be conducted to recognize the tapping point when weaker power plays or opportunistic behavior are not tolerated anymore, and can it terminate relationships?	Chowdhury et al., 2016
3	What could be the roles of resources economic and exchange process actors that remain similar in the B2C context? Future studies should study if the structure of interactions in the B2C system is contingent on the service target as in B2B system?	Breidbach and Maglio, 2016
4	In-depth research is needed for institutional theory application and to check out whether institutional structure affects participating organizations or not, and to remove the gap between S-D logic and institutional approach. We also need to study VCC in the services ecosystem, service value system, and service network.	Kohtamäki and Rajala, 2016
5	Who are effective managers, and what are the leadership styles that make them effective? We also urge future researchers to study the role of customers’ engagement intensity in different phases of the solution process.	Petri and Jacob, 2016
6	Further investigations should focus on the contemporary contributions of all stakeholders to the VCC process and their role in B2B2C markets.	Mingione and Leoni, 2020
7	Future research should develop a process model on how platforms foster the standardization of VCC practices and how it emerges.	Hein, 2019
8	Future research should focus on networks where different actors have more congruent goals and aligned interests and explore how and when SBPs result in synergies and enhanced value outcomes between network actors. For future research, an interesting avenue would be to employ longitudinal research setting and explore how different tensions grow and spread over networks and how individual and collective actors try to mitigate or avoid them over time.	Tura et al., 2019
**Cluster 2**		
9	Future research should focus on gathering data from multiple stakeholders, including multiple informants, on saving and utilizing this data in building case studies to get findings to maximize reliability.	Singaraju et al., 2016
10	We urge future research to find a way to track interactions among resources systematically at times as a service engagement unfolds.	Stucky et al., 2011
11	Future studies should investigate which of the brand-related determinants are unimportant for B2B brand communities?	Bruhn et al., 2014
12	There is a dire need to decrypt more mechanisms underlying the relationship between customer VCC and CSR. Other mediation mechanisms should be considered, especially at the team level, such as team service or customer learning.	Tuan et al., 2019
**Cluster 3**		
13	The conceptual model’s associations should be explored across various B2B sectors and compare its characteristics, drivers, inhibitors, and relative significance. Research could quantify the outcome of proposed conceptual associations across B2B customer segments that reflect varying strategic important levels for the supplier.	Hollebeek, 2019
14	Researchers should study the effect of actors’ opportunism in B2C and open innovation contexts. Future studies could investigate our finding that value is co-destroyed by multiple actors in different sectors. We also need to understand that alliances succeed despite actors’ motivation to minimize transaction costs, using other theoretical viewpoints like dynamic capability.	Pathak et al., 2020
**Cluster 4**		
15	As VCC is considered the outcome of tangled constructs, we should explore their potential influence to have a complete picture and understand all involved processes.	Baumann and Le Meunier-FitzHugh, 2015
16	We urge future research to extend our existing proposed framework by examining customer value assessment in the broader set of B2B firms and generalizing customer value assessment theory in B2B markets.	Keränen and Jalkala, 2013
17	Future research should focus on value co-destruction and, therefore, build on Ostrom et al.’s (2015, p. 133) research priorities on services literature: “to understand VCC negative consequences.” Longitudinal studies should be conducted in which companies’ co-creation experiences come from developing strong ties based on trust and mutual commitment over time.	Berenguer-Contrí et al., 2020

The research guidelines suggested by the first cluster highlighted concepts, like communication skills and dynamic capabilities, leadership and management role, and customer value. The future research direction from Cluster 1 urged future research to focus on the concepts mentioned above and find their association with VCC (Marcos-Cuevas et al., 2016; Petri and Jacob, 2016). VCC is a new field, and along with B2B research should also focus on B2C (Breidbach and Maglio, 2016). Kohtamäki and Rajala (2016) focused on service-dominant logic and urged to focus on bridging the gap in SD-Logic and institutional approach. Cluster 2 emphasized concepts like organizational and dynamic capabilities and the role of management in utilizing these capabilities to create and enhance VCC. It also focused on the knowledge management application effectiveness in marketing and on establishing and developing systems perspective, utilization of big data in multi stake holder systems (Singaraju et al., 2016), and the role of CSR in VCC (Tuan et al., 2019).

Cluster 3 is about how the performance, trust, commitment, interaction, and relationship between buyer and seller (producer and customer) create and augment VCC. It endorses future research to focus on how to engage customers, actors, buyers, and stakeholders in a way that improves their interaction among the parties to enhance VCC and performance. Cluster 4 was the smallest cluster; however it mostly focused on the process of VCC and urges future researches to focus on extending the existing framework for VCC in B2B (Keränen and Jalkala, 2013), how does value co-destruction work (Berenguer-Contrí et al., 2020) as shown in Table 11. Appendix Table 1 in the appendix section at the end of the paper show the future research guidelines of some research papers. Despite showing low citations these research papers are still important based on their relevance and nature of research work.

## Conclusion

Our research article contributed to the understanding of the literature on VCC in the B2B context by grouping the articles into clusters systematically and exploring future research streams. Our study identified influential authors, articles, journals, institutions, and countries through HistCite, which contributed to VCC research in the B2B context. We received 138 publications in “VCC” in “B2B” September (2022). We refined the publications to 121 by selecting articles from Business, management, operations research management science, and social sciences interdisciplinary. Furthermore, we categorized the top fifty-six research articles into four clusters through VOS viewer’s cartographic analysis. We studied each cluster in-depth through content analysis and proposed future research guidelines as well. Our research work will guide researchers in the marketing discipline to investigate various aspects of VCC and determine the research work’s growth by title, topic, measurement, and context.

### Theoretical implications

This study was assisted by bibliometric analysis to provide meaningful insight for other researchers. From content analysis, we have achieved certain novel aspects and understanding about this concept. Some scholars believe that VCC is still an ambiguous concept, and scholars have a difference of opinion regarding VCC’s nature and modality. Existing models of VCC in B2B perspectives purposed by Chen and Nath (2004), Prahalad and Ramaswamy (2004b), Andersson et al. (2007) provide a wide range of conceptual differences. A group of scholars believes that further research is needed to bridge the gap between the new institutional approach and S-D logic. They urge, that there is a need for studies of VCC in the service network, service ecosystem, and service value system (Kohtamäki and Rajala, 2016). Another group of scholars believes, that current studies of customer-based VCC processes in multiple stakeholders environments focus on CB relationships (Cova et al., 2011; Merz et al., 2018). While only a few researchers have tried to investigate how customer-based value is co-created in the industrial environment (Gupta et al., 2017; Törmälä and Saraniemi, 2018; Iglesias et al., 2020). So, another venue of future academia could focus on how CB relationships co-create value in B2B interactions. While a group of authors believes that despite practitioners’ (Accenture, 2014, 2016) and scholars’ attention (Brotspies and Weinstein, 2019; Iankova et al., 2019), nothing is known on how value is co-created in customer-based A2A interactions and multiple stakeholders in B2B2C markets. Moreover, Iankova et al. (2019) call for future research that a wealth of research opportunities exist in B2B2C models.

### Practical implication

Value co-creation research in the B2B environment is in the incubation stage; as presented by bibliometric and content analysis, this study reveals that most papers were published in the last decade specifically post-2018. With web 2.0 rise, business firms can easily achieve a competitive advantage, market share, and VCC (Cova and Cova, 2012; Shah et al., 2021b). Organizations can involve customers in the value creation process to add desired features and changes to products and services to increase their value and organizational value, eventually leading to performance and competitive edge. According to Campbell (2003), Harkison (2018), Wu et al. (2018), organizations gain knowledge from specific customers and then utilize this information to gain competitive advantage through delivering a unique and personalized set of services. Thus, VCC can help get a competitive advantage if organizations learn to manage the process correctly (Prahalad and Ramaswamy, 2004b; Navarro et al., 2015; Oyner and Korelina, 2016; Harkison, 2018).

### Limitations and future research

From content analysis, we learned that VCC is a new construct, especially in the B2B context, and both have their significance in today’s business environment. Our first limitation is that we did not study VCC in other multi-stake holder environment (B2C and B2B2C context); so, we recommend future research to study VCC in the B2C and B2B2C context. Second, in this study, for content analysis, we ignored articles having less than 10 citations during cluster and co-citation analysis. Thus, the recently published research papers were not considered for contribution as their citation index was less than 10.92 out of 121 research articles were published in the past 5 years after 2018 which is 76 percent of the total, which shows the novelty of the concept and is a hot research area. Apriliyanti and Alon (2017) argue that it is essential to conduct bibliometric analysis every 5 years or once in 10 years so that emerging research constructs are highlighted. Thirdly, this study ignored research articles from sources other than WoS. Questions may be raised on our research paper for preferring high-quality publications and ignoring non-WoS journal articles. We recommend future researchers to study research articles from SCOPUS and other data bases as well. Future researchers should also use other Bibliometric software tools to analyze non-WoS journal articles related to VCC in the B2B context. However, this study possesses novel research insights into VCC in B2B contexts for research academia.

As some scholars believe that value and VCC are still ambiguous concepts and there lies a difference of opinion regarding VCC’s nature and modality, so we urge future research to establish some sort of accord. Relationships and its precursors are vital resources and aspects that when efficiently utilized by organizations can give fruitful results in enhancing brand loyalty, customer retention, VCC, competitive edge, and organizational performance. However, there is also a lack of empirical studies which can show us how CB value is co-created in multiple stakeholder and industrial environments (B2B, B2C, and B2B2C). There is a dire need for future studies to focus on how business relationships could be best utilized for co-creating costumer and organizational-based value in B2B interactions. We also urge future research to put an effort in bridging the gap between the service-dominant logic and new institutional approach. The existent proposed models by various researchers within VCC in B2B have a wide range of differences among them, we request future research to propose a model which in unanimously accepted. Achieving VCC is not the ultimate target, for achieving a long-term competitive edge future research need to find a way how can organizations make the process of VCC both sustainable and economically viable.

With the discussion, we have made in Sections “Research guidelines for future studies” and “Limitations and future research” we have answered our fourth and final research question (RQ4).

## Author contributions

All authors listed have made a substantial, direct, and intellectual contribution to the work, and approved it for publication.

## Appendix

**APPENDIX TABLE 1:** Future research guidelines of some important articles.

Sr no	Future research guidelines	Authors
1	This study aimed to proceed with the KM perspective on marketing; still, a lope hole for future research exists in applying the SBKM method to improve marketing effectiveness through KM perspectives both in B2B and broader applications?	Powell and Swart, 2010
2	What kind of communicative skills are required by CSRs in a non-cooperative interaction with consumers that can support value co-creation.	Salomonson et al., 2012
3	We urge future researchers to quantify our framework’s elements of the socio-cognitive construct of customer value with influence tactics.	Hohenschwert and Geiger, 2015
4	What is the difference between the customer and consumer role? Future studies should differentiate among the concept of VCC, value proposition, and co-creation of services based on value proposition?	Muzellec et al., 2015
5	We believe many business networks studies do not capture the multiple perspectives of the involved stakeholders or actors (cf. Aarikka-Stenroos et al., 2014). ecosystem layer application increases the need to involve multiple perspectives and study their diverse interactions, ontology, and complicated methods of research (Spohrer, 2011; Leroy et al., 2013).	Aarikka-stenroos and Ritala, 2017
